# Annual variation in daily light exposure and circadian change of melatonin and cortisol concentrations at a northern latitude with large seasonal differences in photoperiod length

**DOI:** 10.1186/s40101-016-0103-9

**Published:** 2016-07-19

**Authors:** Mathias Adamsson, Thorbjörn Laike, Takeshi Morita

**Affiliations:** School of Engineering, Jönköping University, P.O. Box 1026, SE-551 11 Jönköping, Sweden; Department of Architecture and Built Environment, Lund University, P.O. Box 118, SE-221 00 Lund, Sweden; Department of Environmental Science, Fukuoka Women’s University, 1-1-1 Kasumigaoka, Higashi-ku, Fukuoka, Japan

**Keywords:** Light exposure, Spectral composition, Circannual, Northern latitude, Melatonin, Cortisol

## Abstract

**Background:**

Seasonal variations in physiology and behavior have frequently been reported. Light is the major zeitgeber for synchronizing internal circadian rhythms with the external solar day. Non-image forming effects of light radiation, for example, phase resetting of the circadian rhythms, melatonin suppression, and acute alerting effects, depend on several characteristics of the light exposure including intensity, timing and duration, spectral composition and previous light exposure, or light history. The aim of the present study was to report on the natural pattern of diurnal and seasonal light exposure and to examine seasonal variations in the circadian change of melatonin and cortisol concentrations for a group of Swedish office workers.

**Methods:**

Fifteen subjects participated in a field study that was carried out in the south of Sweden. Ambulatory equipment was used for monthly measurements of the daily exposure to light radiation across the year. The measurements included illuminance and irradiance. The subjects collected saliva samples every 4 h during 1 day of the monthly measuring period.

**Results:**

The results showed that there were large seasonal differences in daily amount of light exposure across the year. Seasonal differences were observed during the time periods 04:00–08:00, 08:00–12:00, 12:00–16:00, 16:00–20:00, and 20:00–24:00. Moreover, there were seasonal differences regarding the exposure pattern. The subjects were to a larger extent exposed to light in the afternoon/evening in the summer. During the winter, spring, and autumn, the subjects received much of the daily light exposure in the morning and early afternoon. Regarding melatonin, a seasonal variation was observed with a larger peak level during the winter and higher levels in the morning at 07:00.

**Conclusions:**

This study adds to the results from other naturalistic studies by reporting on the diurnal and seasonal light exposure patterns for a group living at a northern latitude of 56° N, with large annual variations in photoperiod length. It seems to be seasonal variation in the lighting conditions, both concerning intensities as well as regarding the pattern of the light exposure to which people living at high latitudes are exposed which may result in seasonal variation in the circadian profile of melatonin.

## Background

Seasonal variation in various characteristics of physiology and behavior has frequently been reported in modern-day humans [1–9]. Light is the major zeitgeber, or time cue, for synchronizing internal near 24-h rhythms to the temporal change of the external light and dark cycle [10–12].

Action spectra for the melatonin suppression response and pupillary constriction response in humans have identified the peak sensitivity of the circadian system between 440 and 482 nm [13–15]. Several non-image forming functions are similarly more sensitive to short wavelength monochromatic light radiation than monochromatic light radiation in the middle and long wavelength range [16–22]. Moreover, polychromatic light enriched in the short wavelength part of the spectrum resulted in a larger suppression of melatonin in comparison to polychromatic light with a lesser content of short wavelength light [23, 24].

Timing, duration, intensity, spectral composition, and previous light exposure determine the non-image forming response to light. Phase response curves (PRCs) are consistent in showing that light exposures in the early biological night induces phase delays and light exposures during the late biological night/early morning phase advance the circadian clock [25–29]. Light exposures during the day have reduced, but not insignificant, effects on the phase position of the circadian pacemaker [30]. The amplitude of phase shifts are dose dependent and non-linear [31, 32]. Moreover, exposure to 6.5 h of 480-nm monochromatic light, although containing 4 % of the energy from a 6.7-h pulse of polychromatic light, elicited 75 % of the phase resetting response of the pulse of polychromatic light pulse [33].

Studies have shown that ordinary room light is capable of eliciting half of the maximum response as evidenced by phase resetting of the circadian rhythms of melatonin, cortisol and body temperature, acute suppression of nocturnal melatonin secretion, and subjective and objective measures of alertness [34, 35].

Results from laboratory experiments and studies conducted in natural conditions have shown a greater suppression of nocturnal melatonin and a greater phase resetting response to light exposure following an exposure to dim light or darkness than after a prior exposure to higher light intensities, indicating that humans may display a seasonal variation in sensitivity to melatonin suppression and phase shifting of the melatonin rhythm [36–40]. Higuchi et al. [41] found that higher amount of daytime light exposure in the summer resulted in a lower melatonin suppression by nocturnal light exposure and Morita et al. [42] demonstrated differences in the phase of the circadian melatonin rhythm depending on levels and pattern of light exposure. These results are supported by other studies showing an increased nocturnal secretion of melatonin secretion and a reduced suppressing effect of evening light following exposure to bright light during the daytime [43–46]. In addition, Kozaki et al. [47] found that exposure to short wavelength enriched light in the morning was more effective in preventing melatonin suppression by nighttime light in comparison to morning exposure to light with a lower correlated color temperature, containing a lower amount of short wavelength light.

Laboratory experiments have shown that humans maintain a capacity to adjust physiology and behavior to different photoperiod lengths [48–50]. Results from studies conducted under natural conditions in locations with considerable seasonal variation in photoperiod length have shown seasonal variations in duration of nocturnal and daily melatonin secretion with longer durations in the winter [51, 52] and, in melatonin peak amplitude, daily and nocturnal concentrations of melatonin with higher levels in the winter in comparison to the summer [53–56] and, in phase position of circadian rhythms of melatonin and cortisol [57, 58] with an advanced phase in summer and autumn while others have found no differences in phase position between summer and winter [41, 59]. A study by Matthews et al. [60] showed no seasonal variations in nocturnal and diurnal concentrations of melatonin. Moreover, Wehr et al. [61] found no seasonal variations in duration of elevated levels of melatonin, cortisol, and thyrotropin and low levels of body temperature. Interestingly, the melatonin rhythm was more closely synchronized to the sleep-wake cycle and exposures to artificial light than to the natural photoperiod. Others have demonstrated a delayed phase position of the melatonin rhythm in spring and summer in comparison to winter due to an increased exposure to evening light [56, 62]. The description of seasonal affective disorder (SAD) and the milder non-clinical form subsyndromal SAD (S-SAD) has led to hypothesis involving the circadian pattern of melatonin secretion in the pathogenesis of the condition [63]. Studies investigating circadian profiles of rhythms generated by the endogenous circadian pacemaker in SAD and normal controls have indicated a delay relative to the sleep-wake cycle during the depressed episodes while other studies have found no seasonal misalignment in SAD or differences in the circadian profile between healthy subjects and SAD patients [64–71]. Salivary cortisol concentrations were reported to be highest in February, March, and April and lowest in July and August [72]. On the other hand, the results from a study by Hansen et al. [73] showed highest cortisol levels during the autumn/winter period. Scheer et al. [74] reported increases following light exposure in the morning while Kostinouglou et al. [75] showed decreases after bright light exposure in the evening and Jung et al. [76] reported a suppressing effect of light when administered during both the ascending and descending phase of circadian rhythm of cortisol.

Field studies investigating the diurnal and seasonal pattern of light exposure report of significantly higher exposure to bright light (i.e., exposure to illuminance exceeding 1000 lux) in the summer compared with the winter and this difference is more marked at higher latitudes. Furthermore, it was shown that most subjects spent a majority of the waking day in ordinary room intensities less than 1000 lux, in both summer and winter [77–79]. The light exposure typically increases from the morning reaching a peak in late morning/early afternoon [6, 80, 81]. Thorne et al. [80] found a higher light exposure in summer than winter and also demonstrated seasonal differences with respect to spectral composition such that the subjects were exposed to a higher ratio of short wavelength light, especially in the evening, during the summer. Similarly, Figueiro et al. [62] reported that adolescents were exposed to significantly more photopic and circadian light in the spring than winter, particularly in the evening which was attributed to an increase of exposure to natural light rather than to seasonal differences in the use of artificial light. Most field studies conducted under natural conditions suggest that the human circadian pacemaker is continuously entrained primarily by intermittent bright light pulses distributed throughout the day [78, 79]. Hebért et al. [78] suggest that weak non-photic zeitgebers, for example, arousal, the sleep-wake cycle, and other social cues are able to entrain the circadian pacemaker in combination with a moderately bright light-dark cycle.

The aim of the present study was to report on the natural pattern of diurnal and seasonal illumination to which a group of office working people in Sweden normally are exposed in their real working and home environments. Another goal was to investigate if there is a seasonal variation in the circadian change of melatonin and cortisol concentrations. It was hypothesized that the subjects would show seasonal differences in the timing, intensity, and spectral composition of the exposing light radiation as well as in the circadian change of melatonin and cortisol concentrations. First of all, during the summer, the subjects would be exposed to more light radiation in comparison to the other seasons. Secondly, a seasonal variation in the pattern of exposure was expected. Thirdly, higher levels of melatonin were anticipated during the winter in addition to a delayed circadian rhythm of melatonin. Finally, the levels of cortisol were hypothesized to show an increase during the spring.

## Method

### Sample

The study was carried out in the south of Sweden (56° N) from February 2008 to January 2009. Fifteen subjects, 13 women and 2 men (mean age = 46.1 years, SD = 9.8, range 28–61 years), were recruited to participate in the study. The study was approved by the ethics committee at Fukuoka Women’s University. All subjects gave informed consent after attending a seminar where the purpose, described as general environmental impact, and procedure of the study was explained. It was stressed that participation was voluntary and that the subjects could withdraw at any time. The collection of data was confidential and it was not possible to identify any individual persons in the data treatment. The subjects were daytime office workers. A typical workweek comprised 36–40 h during weekdays and the subjects started work between 07:00 and 08:10 and ended working between 15:00 and 17:30. The offices were lit by a combination of daylight and fluorescent lamps and the home environments were mainly lit by daylight and incandescent lamps.

### Drop-out

Technical problems with some of the prototype instruments resulted in 16 % missing data from the irradiance recordings. Due to a limited number of available Actiwatch-L monitors, a late change of experimental week by one of the subject resulted in 2 % missing data from the illuminance recordings. Insufficient amount of saliva and errors in analyses resulted in 9 and 13 % missing data from the measurements of melatonin and cortisol, respectively. Missing data was replaced by subjective seasonal mean for the corresponding time period or time point.

### Instruments for ambulatory measurements of light exposure

Two types of instruments for ambulatory recordings of light exposure during daily life were used in the study.

Actiwatch-L (Minimitter/Respironics, Bend, OR) monitors were used to measure exposing illuminance every minute. This is an instrument that has been frequently used in field studies investigating light exposure and sleep-activity rhythms [77, 82, 83].

An additional prototype instrument was used to measure the spectral composition of the exposing irradiance. It had seven channels and a bandwidth of 50 nm, ranging from 400 to 750 nm. The instrument was designed using photonic devices (Hamamatsu Photonics K.K.) and included a linear variable band pass filter (Edmund Optics Inc.) for spectral filtering.

In order to validate the spectral sensitivity, accuracy, and linearity of the instrument, the calibration equations for the seven channels were calculated by comparisons of recorded data with data simultaneously measured with a spectroradiometer (Light Spex: McMahan Research Laboratories) under various daylight and artificial lighting conditions.

The sensor was positioned on the chest and the data storage module was carried in a shoulder bag.

### Procedure

The subjects completed a monthly 3-day experimental period, beginning at 12:00 on Tuesday and ending at 12:00 on Friday.

The subjects were instructed to wear the Actiwatch-L monitors outside the clothing, on the non-dominant wrist, except when bathing from Tuesday 12:00 to Friday 12:00. The portable instrument used for measuring the spectral composition was worn from Wednesday 12:00 to Friday 12:00. Both instruments were placed close to the subjects, for example, on a bedside table, during the night.

During one 24-h period between the third and fourth day of experimental period, the subjects collected saliva at six times; 11:00, 15:00, 19:00, 23:00, 03:00, and 07:00 for assessment of day- and nighttime concentrations of melatonin and cortisol. The subjects were instructed to place the cotton swab in the mouth until it was completely hydrated and the samples were immediately put in a freezer. Furthermore, the subjects were instructed not to drink any alcohol during the experimental period and not to eat, consume coffee or sour liquids, for example, juice, and brush the teeth later than 90 min prior to saliva collection.

At the beginning of the experimental week, the experimenter distributed the recording instruments, saliva tubes, and a weekly log containing daily sleep-activity diaries where the subjects graphically reported their wake time, bedtime, and time spent outdoors and at work. At the end of the experimental period, the recording instruments and weekly log were collected by the experimenter at the subjects’ workplaces.

Recordings of integrated amount of exposing illuminance and irradiance, calculated by adding the values that were recorded every minute, were divided into six 4-h time periods during the day; 00:00–04:00, 04:00–08:00, 08:00–12:00, 12:00–16:00, 16:00–20:00, and 20:00–24:00 and averaged across the days of measurement. The irradiance data were categorized into five wavelength ranges, short wavelength light (400–550 nm), middle wavelength light (550–650 nm), long wavelength light (650–750 nm), total irradiance (400–750 nm), and circadian wavelength (450–500 nm).

Existing human phase response curves show slightly different inflection points where the direction of the responses changes from phase advances to phase delays [23–27, 31]. In this study, a time period from 04:00 to 12:00 was chosen for phase advances of the endogenous circadian pacemaker and phase delays were estimated to occur from 18:00 to 04:00. Between 12:00 and 18:00, small changes in phase position in response to light exposure were expected. Therefore, relative light exposure during these time periods were estimated in order to relate the daily light exposure to possible effects on the phase of the endogenous circadian pacemaker.

### Analysis of melatonin and cortisol

Commercial ELISA kits (Buhlmann Laboratories AG Swiss) were used for the analysis of salivary melatonin and cortisol. Peak time and peak levels of melatonin were calculated by spline interpolation of the original points [84]. Intra-assay precision (within-run) was 12.6 %. The intra-assay precision was calculated from the results of four different saliva samples within the standard range, measured 10 times in duplicate in a single run. Inter-assay precision (run-to-run) was 22.9 %. The inter-assay precision was calculated from the results of 17 independent runs with 5 samples within the standard range.

### Data analysis

The Statistical Program for Social Sciences, SPSS, version 19, was used for the analyses. Seasonal differences in peak level and peak time of the melatonin rhythm were analyzed by means of a 1 × 4 repeated measures ANOVA and the results are reported as group means and standard deviations.

Moreover, seasonal differences concerning concentrations of melatonin were also analyzed by a 6 × 4 repeated measure ANOVA with “time point” and “season” as independent variables and “melatonin concentration” as the dependent variable. The results are reported as group means and standard deviations.

Seasonal variations in cortisol concentrations were analyzed by comparing the concentration at the six time points during the day by means of a 6 × 4 repeated measures ANOVA with “cortisol concentration” as dependent variable and “time point” and “season” as independent variables. The results are reported as group means and standard deviations.

Seasonal variation in the exposing illuminance and irradiance was analyzed by a 6 × 4 repeated measures ANOVA with the two factors “time period” and “season” as independent variables and illuminance and irradiance as dependent variables and are reported as group means and standard deviation.

A *p* value of <0.05 was considered to be significant.

The months were divided into seasons with respect to photoperiod such that winter included November, December, and January; spring included February, March, and April; summer included May, June, and July; and autumn included August, September, and October.

## Results

**Fig. 1:**
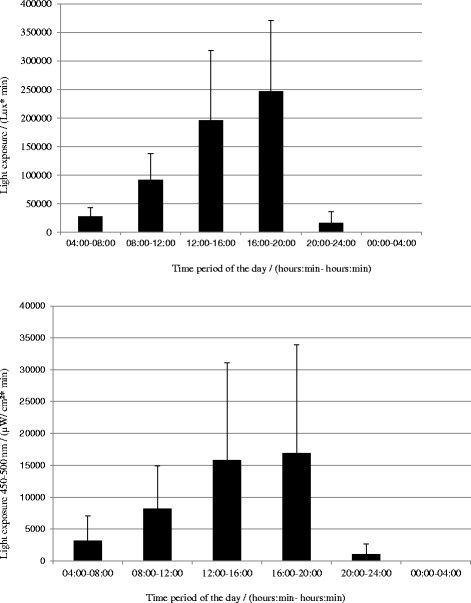
Annual integrated exposure (group mean ± SD) to illuminance and irradiance in the range 450–500 nm during the six time periods of the day

All the results were measured both as illuminance and irradiance. The correlation between the two measurements were *r* = 0.78, which is a fairly good correlation. Analysis of daily light exposure patterns showed a diurnal variation in light exposure with very low values during the nighttime (Fig. 1). Also, see Table 1 for reports of means and standard deviations.

**Table 1:** Means and standard deviations for annual integrated exposure to illuminance and irradiance in the range 450–500 nm during the six time periods of the day

		Time period of the day (hh:mm–hh:mm)					
		04:00–08:00	08:00–12:00	12:00–16:00	16:00–20:00	20:00–24:00	00:00–04:00
Light exposure	Mean (lux*min)	27,654	91,945	196,408	246,982	16,621	166
*N* = 15	SD (lux*min)	15,922	46,024	121,967	124,183	19,830	305
Light exposure 450–500 nm	Mean (μW/cm^2^*min)	3166	8226	15,791	20,162	1122	20
*N* = 14	SD (μW/cm^2^*min)	3925	6742	15,143	20,966	1799	41

**Fig. 2:**
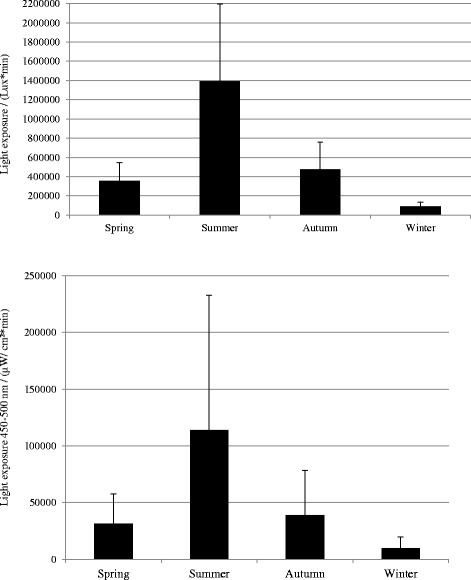
Integrated exposure (group mean ± SD) to illuminance and irradiance in the range 450–500 nm across the seasons

Figure 2 displays the difference in light exposure across the seasons (Also, see Table 2 for reports of means and standard deviations). The subjects were exposed to more light radiation during the summer in comparison to the winter, spring, and autumn (*F*(3,39) = 12.58, *p* < 0.001). Contrasts revealed that the daily light exposure during the summer was significantly larger than autumn (*F*(1,14) = 42.24, *r* = 0.87), spring (*F*(1,14) = 36.78, *r* = 0.85), and winter (*F*(1,14) = 66.60, *r* = 0.91).

**Table 2:** Means and standard deviations for integrated exposure to illuminance and irradiance in the range 450–500 nm across the seasons

		Season			
		Winter	Spring	Summer	Autumn
Light exposure	Mean (lux*min)	91,366	356,368	1,394,200	477,165
*N* = 15	SD (lux*min)	41,984	190,282	800,295	280,363
Light exposure 450–500 nm	Mean (μW/cm^2^*min)	9919	31,153	114,027	38,846
*N* = 14	SD (μW/cm^2^*min)	9590	26,400	118,749	39,721

However, when comparing each season with the other, it was revealed that the light exposure did not differ between spring and autumn. Analyses of the irradiance measurements showed that the subjects were exposed to significantly more light radiation in the range 450–500 nm during the summer compared to the winter, spring, and autumn (*F*(3,39) = 12.58, *p* < 0.001). Contrasts revealed that the daily light exposure in wavelength range between 450 and 500 nm during summer was significantly larger in the summer in comparison to the autumn (*F*(1,13) = 13.80, *r* = 0.72), spring (*F*(1,13) = 9.80, *r* = 0.66), and winter (*F*(1,13) = 14.81, *r* = 0.73).

**Fig. 3:**
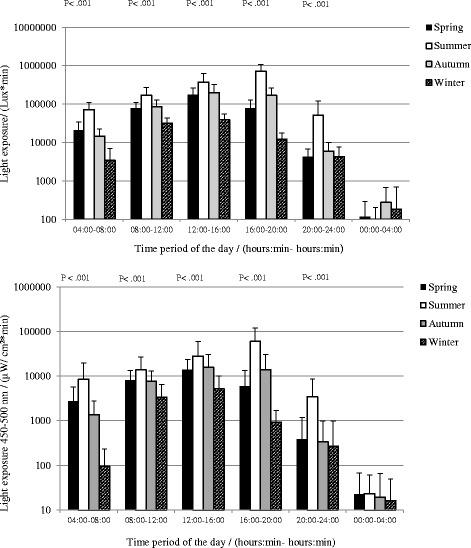
Seasonal integrated exposure (group mean ± SD) to illuminance and irradiance in the range 450–500 nm during the six time periods during the day

Similar to the measurements of exposing illuminance, no significant difference in the total daily irradiance in the range 450–500 nm was observed between spring and autumn. A significant seasonal difference was observed during all time periods except during nighttime (see Fig. 3). Also, see Table 3 for reports of means and standard deviations. Furthermore, it was quite large variations within the subject group as can be seen in the SDs.

**Table 3:** Means and standard deviations for seasonal integrated exposure to illuminance and irradiance in the range 450–500 nm during the six time periods during the day

Season		Time period of the day (hh:mm–hh:mm)					
		04:00–08:00	08:00–12:00	12:00–16:00	16:00–20:00	20:00–24:00	00:00–04:00
Winter	Mean (lux*min)	3499	31,829	39,325	12,227	4304	182
*N* = 15	SD (lux*min)	3573	12,142	16,712	5697	3335	524
Spring	Mean (lux*min)	20,848	77,887	174,058	79,184	4275	116
*N* = 15	SD (lux*min)	13,858	31,940	91,305	50,529	2474	176
Summer	Mean (lux*min)	71,518	172,843	371,683	726,036	52,033	88
*N* = 15	SD (lux*min)	38,046	95,915	252,050	344,795	69,369	119
Autumn	Mean (lux*min)	14,753	85,219	200,564	170,479	5873	277
*N* = 15	SD (lux*min)	8210	44,098	127,800	95,712	4141	402
Winter	Mean (μW/cm^2^*min)	97	3386	5238	916	267	16
*N* = 14	SD (μW/cm^2^*min)	136	3142	4777	782	719	34
Spring	Mean (μW/cm^2^*min)	2718	8157	14,023	5850	384	22
*N* = 14	SD (μW/cm^2^*min)	2990	5357	9833	7371	802	46
Summer	Mean (μW/cm^2^*min)	8503	13,762	28,070	60,170	3500	23
*N* = 14	SD (μW/cm^2^*min)	11,181	12,943	30,928	58,643	5016	37
Autumn	Mean (μW/cm^2^*min)	1347	7598	15,834	13,711	337	20
*N* = 14	SD (μW/cm^2^*min)	1392	5525	15,032	17,066	660	46

**Fig. 4:**
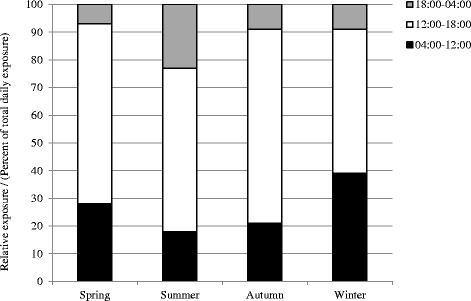
Relative integrated mean illuminance exposure during the day across the seasons

Figure 4 shows that during the winter, the subjects received on average 39 % of the daily light exposure between 04:00 and 12:00 and 9 % from 18:00 to 04:00. During the autumn, the subjects received on average 21 % of the daily light exposure between 04:00 and 12:00 and 9 % during the time period between 18:00 and 04:00. The spring showed a similar light exposure pattern with 28 % of the daily light exposure between 04:00 and 12:00 and 7 % during evening/night. In the summer, the subject group received on average 18 % of the daily light exposure from 04:00 to 12:00 and 23 % from 18:00 to 04:00.

**Fig. 5:**
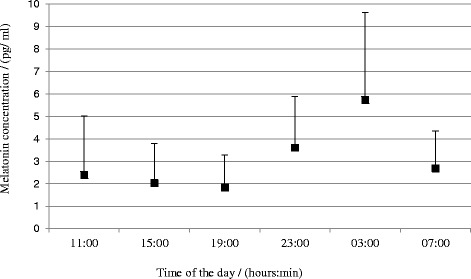
Annual mean melatonin concentration at the six time points during the day

**Fig. 6:**
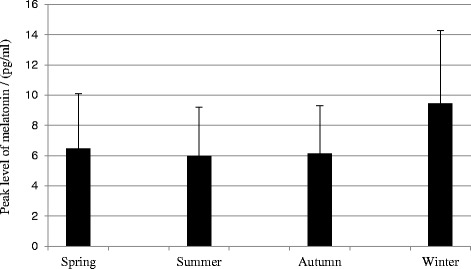
Peak level of melatonin across the seasons as derived from the SPLINE analysis

Turning to the measurements of the biological markers: The melatonin profile showed a diurnal variation (see Fig. 5). Also, see Table 4 for reports of means and standard deviations. Furthermore, Fig. 6 illustrates that there was a seasonal variation in peak level of melatonin with higher concentrations in the winter than in the spring, summer, and autumn (*F*(3,42 = 5.67, *p* = 0.002)). Also, see Table 5 for means and standard deviations. Contrasts showed that during the winter, the peak level of melatonin was higher in the winter than in the spring (*F*(1,14) = 6.87, *r* = 0.57), summer (*F*(1,14) = 7.07, *r* =0 .57), and autumn (*F*(1,14) = 7.07, *r* = 0.57).

**Table 4:** Means and standard deviations for seasonal and annual mean melatonin concentration at the six time points during the day

Season		Time point (hh:mm)					
		11:00	15:00	19:00	23:00	3:00	7:00
Winter	Mean (pg/ml)	3.12	2.30	1.94	4.52	6.74	3.82
	SD (pg/ml)	4.59	2.51	1.47	2.95	5.15	1.91
Spring	Mean (pg/ml)	2.20	2.06	1.66	3.89	5.44	2.30
	SD (pg/ml)	1.56	1.76	1.06	2.04	3.84	1.53
Summer	Mean (pg/ml)	2.09	1.95	1.81	3.15	5.97	2.32
	SD (pg/ml)	1.74	1.31	1.62	2.05	3.68	1.53
Autumn	Mean (pg/ml)	2.12	1.91	1.88	3.03	5.24	2.47
	SD (pg/ml)	1.54	1.51	1.64	1.68	3.83	1.21
Annual average	Mean (pg/ml)	2.39	2.03	1.84	3.61	5.73	2.69
	SD (pg/ml)	1.47	1.38	1.50	3.34	5.08	2.58

**Table 5:** Means and standard deviations for peak level of melatonin across the seasons as derived from the SPLINE analysis

		Season			
		Winter	Spring	Summer	Autumn
Peak level of melatonin	Mean (pg/ml)	9.44	6.48	5.97	6.13
*N* = 15	SD (pg/ml)	4.82	3.60	3.22	3.19

Comparisons between concentrations at the different time points during the day showed that the concentration at 07:00 was significantly higher during the winter (*F*(3,39) = 5.59, *p* = 0.003). No seasonal variation in peak time of melatonin secretion was observed.

**Fig. 7:**
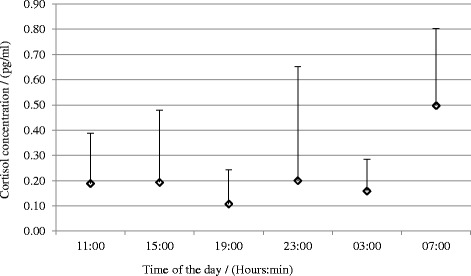
Annual mean cortisol concentration at the six time points during the day

Figure 7 shows the cortisol profiles over the day with the typical high peak in the morning (See Table 6 for reports of means and standard deviations).

**Table 6:** Means and standard deviations for annual mean cortisol concentration at the six time points during the day

		Time of the day (hh:mm)					
		11:00	15:00	19:00	23:00	3:00	7:00
Cortisol concentration	Mean (pg/ml)	0.19	0.19	0.11	0.20	0.16	0.50
*N* = 15	SD (pg/ml)	0.20	0.28	0.13	0.45	0.13	0.31

The ANOVA repeated measures analyses of cortisol concentrations at the six time points during the day did not show any seasonal variation.

## Discussion

This longitudinal study investigated seasonal light exposure patterns for a group of Swedish office workers in their working and home environments. To our knowledge, this is the first study reporting seasonal data regarding illuminance and irradiance exposure and melatonin and cortisol secretion measured every month across the year for the same subjects. Moreover, few field studies have been conducted at this latitude (56° N) where there are large seasonal variations in photoperiod, with a difference in day length of more than 10 h between the summer and the winter. This also means that this location is very suitable for investigating seasonal variations in light exposure and physiology. In spring (March 23), the natural photoperiod begins at 06:03 and ends at 18:30; in the summer (June 23), it begins at 04:23 and ends at 22:00; in the autumn (September 23), it begins at 5:57 and ends at 18:05; and in the winter (December 23), it begins at 8:40 and ends at 15:37. Consequently, in the winter, it was generally dark when the participants commuted to and from work. Moreover, there are seasonal differences in temperature, hours of sunshine, and total daily solar radiance.

Most studies using ambulatory recordings of diurnal and seasonal exposure to light radiation have been conducted at latitudes between 32° N and 51° N and only a few have examined the spectral composition of the daily exposure to light radiation.

In this study, both illuminance and irradiance were measured but the placement of the sensors was different. However, after replacing the missing data with individual seasonal average for the corresponding time period, the field measurements showed a fairly high correlation between the instruments [85–88]. Moreover, the use of the two instruments presented a possibility to examine the spectral composition of the exposing light radiation which is of particular interest since the peak sensitivity of the melanopsin containing ipRGCs is shifted towards the short wavelength part of the spectrum.

The seasonal pattern of light exposure showed several differences across the seasons. As hypothesized, the participants received significantly more light radiation during the summer (daily mean = 1,394,200 lux*min) in comparison to the winter (daily mean = 91,366 lux*min), spring (daily mean = 356,367 lux*min), and autumn (daily mean = 477,165 lux*min). This is in line with studies showing significant seasonal difference in exposure to bright light in Montreal, Canada (45° 31′ N) and Rochester, USA (44° 1′ N) with six times more exposure to bright light, exceeding 1000 lux, in the summer compared to the winter [78, 79, 89].

During the summer, the participants were on average exposed to more light radiation during all time periods of the day except during the nighttime and particularly during their leisure time in the afternoon and evening. Similar to the results of Thorne et al. [80], the participants were exposed to more irradiance in the range 450–500 nm in the afternoon/evening during the summer in comparison to winter, spring, and autumn. Moreover, the subjects were exposed to a higher amount of illuminance and irradiance in the range 450–500 nm in the afternoon/evening during the spring and autumn in comparison to the winter. This is in line with the results from Figueiro et al. [62] which report of greater exposure to circadian light in the evening during the spring than the winter for a group of adolescents.

Similar to other studies, the results also showed that the light exposure patterns varied considerably within the group of subjects [78, 79, 90, 91].

In this study, the participants were exposed to a daily average of 7953 lux-hours (range = 3947–15,690 lux-hours) in the autumn, 5939 lux-hours (range = 1662–10,978 lux-hours) in the spring, 23,237 lux-hours (range = 12,741–52,447 lux-hours) in the summer, and 1523 lux-hours (range = 929–2330 lux-hours) in the winter. This can be compared with Hubalek et al. [92] which reported a mean daily exposure of 7394 lux-hours for group of Swiss subjects studied in May–June. Moreover, Oren et al. [93] showed a daily light exposure of 1626 and 1521 lux-hours during the winter for a group of SAD patients and a group of healthy controls. From the results of the present study, it seems that this group of office workers was exposed to similar light exposure levels as people with comparable occupation living at locations nearer the equator during the spring and autumn months. However, during the summer, with long photoperiods with daylight for more than 18 h, they appear to be exposed to more light radiation but in the winter they seem to be exposed to somewhat lesser light radiation in comparison to people living at locations closer to the equator.

Several studies regarding shift work have demonstrated the importance of including the 24-h pattern of light exposure by showing that the timing and contrast between daily light intensities and dark periods determine the phase of entrainment [94–98]. The results from the present study showed a higher ratio between the exposure to light radiation from 04:00 to 12:00, when the response to light stimuli will result in a phase advance of the circadian pacemaker and a suppression of melatonin and the total daily exposure to light radiation during the winter in comparison to the spring, summer, and autumn. On the other hand, during the summer, the ratio between the exposure to light radiation from 18:00 to 04:00, when light stimuli will phase delay the circadian pacemaker and suppress melatonin, and the total daily exposure to light radiation was higher in comparison to the spring, autumn, and winter. A number of previous studies have showed that many modern-day people spend much of their time indoors which underlines the importance of the built environment [77, 78]. In the present study, the subjects were exposed to much of the daily light exposure during the time spent at the work place, especially during the winter but also during parts of the spring and autumn which highlights the importance of the light environment in the workplaces. Furthermore, currently, there is an increase in use of new solid state light sources, both in the home environment as well as in exterior lighting fixtures and indoor environments where people spend their leisure time, resulting in more exposure to radiation in the short wavelength part of the spectrum during the evening. Also, light-emitting eReaders and backlit computer screen with LEDs used during the hours before bedtime have an influence on melatonin suppression, phase delay of the circadian rhythm, and an acute effect on alertness [99]. This suggests that modern-day people that spend a large part of their day indoors will to a higher extent be exposed to light radiation in the short wavelength part of the spectrum in the evening than earlier generations. In order to avoid adverse effects, it is therefore important to acknowledge this as a potential problem and also to focus on increasing the exposure to short wave light radiation during the daytime by an increased use of daylight or by using light sources enriched in the short wavelength part of the spectrum.

The monthly measured melatonin profiles showed high nocturnal concentrations and lower daytime concentrations. As hypothesized, the maximum nocturnal concentrations of melatonin were higher during the winter season when the subjects similarly were exposed to lower levels of visible light radiation, in total daily exposure as well as during the different time periods of the day, suggesting an influence of photo period length and light intensity. The peak level of nocturnal melatonin was higher during the winter compared to spring, summer, and autumn which is in accordance with results reported in the literature [41, 54] but differing from other results showing no seasonal variation in peak melatonin concentration [60]. Furthermore, there was no significant difference in peak levels of melatonin between spring, summer, and autumn.

However, some studies have shown higher nocturnal levels of melatonin after exposure to larger amounts of light exposure during the daytime which may be related to the daily pattern of light exposure and the effect of light history [43–45]. When viewing the relative exposure of the subjects in this study, they received a large part of the daily light exposure in the morning (38 %) and less in the evening during the winter (7 %). This means that they received much of the daily light exposure during the phase-advancing part of the phase response curve for a day-active person and a relatively small amount during the phase-delaying part of the PRC resulting in a well-defined circadian profile of melatonin with a high amplitude. Also, the lower levels of light exposure in the early morning may explain the higher values that were observed during the winter season at 07:00.

In this study, we found no seasonal variation regarding the time of maximum secretion of melatonin. Due to the large seasonal variation of the photoperiod but probably also because of the seasonal differences in temperature leading to more time spent outdoors in daylight during the summer, the subjects were exposed to more evening light in comparison to the other seasons. Several studies in real working and living environments have shown a phase delay of the rhythm of melatonin during the winter in comparison to the summer. However, most of those studies have been carried out at a lower latitude than the present study. Other studies of modern-day people have not found any seasonal variation in the melatonin profile and two studies conducted at higher latitudes have found a phase delay of the melatonin rhythm in spring and summer possibly as a consequence as more exposure to light in the evening.

Moreover, they were similarly exposed to considerably more light during the daytime during the summer. Field studies and investigations carried out in laboratory have reported a lower melatonin suppression after daytime exposure to higher levels of light. Therefore, light history and the pattern of light exposure might explain the results found in this study.

Taken together, the results in this study support earlier results showing that timing and daily pattern of light exposure may be more decisive than the total daily exposure for determining the phase of entrainment.

Contrary to the hypothesis, the analyses of the circadian profiles of cortisol showed no seasonal difference. Some studies have shown higher concentrations during the dark period of the year and lower during the summer [73]. On the other hand, Küller and Lindsten [8] demonstrated a seasonal delay regarding the rise in morning cortisol concentration in school children spending their classes in windowless rooms. Moreover, the time for collection of saliva may also have an impact on the results.

### Limitations and suggestions for further research

This field study carried out in real working and living environments have some limitations. The reliability of a study to investigate the effects of the annual variation in daily light exposure on circadian change of melatonin and cortisol concentrations is limited by the size of the sample which includes 15 subjects. However, the effect sizes regarding seasonal variations in light exposure and in peak levels of melatonin were large. Moreover, the age variation between 28 and 61 years is relatively large and is an advantage but on the other hand are different age groups represented by just a few individuals.

In this study, we were interested in investigating the expressed rhythm of daytime and nocturnal concentrations of melatonin and cortisol. The method used, where saliva was collected every 4 h, offered a possibility to estimate the expressed circadian change of melatonin secretion. However, the design of the study made it difficult to control for lighting conditions during the collection of saliva. Saliva samples were collected in the participants’ natural environment and are therefore influenced by masking responses evoked by environmental and behavioral stimuli. During nighttime, low illuminances and irradiance levels, on average between 0.36 and 1.15 lux, were recorded during all seasons. The subjects were on average exposed to higher levels of illuminance in the summer (298 lux) than in the winter (15 lux) in the time period between 04:00 and 08:00 am. This indicates that due to season, the subjects may have been exposed to early morning light through windows which could have impacted the melatonin concentration measured at 07:00. Furthermore, the exposure to evening light, 20:00–00:00, increased by a factor 12 between winter (on average 18 lux) and summer (on average 217 lux), possibly impacting melatonin suppression measured at 19:00 and 23:00. Although we found no seasonal differences when using the present method, the limited number of measuring points and the influence of masking, both by light exposure and by behavioral stimuli, suggests further studies.

The dim light melatonin onset (DLMO) is a more precise marker for estimating the unmasked phase position of the circadian pacemaker [100]. Furthermore, Lewy et al. [101] suggest that by using goggles that blocks short wavelength radiation [102], saliva sampling for assessment of the DLMO could be performed in the subjects’ home environment.

Cortisol is influenced by other factors than light. Jung et al. [76] report of a variety of factors including, for example, stress, physical activity, and sleep loss that can have temporal influence on the diurnal rhythm of cortisol. Furthermore, additional sampling points and thereby a higher resolution would have permitted an estimation of the awakening cortisol response (ACR) which may have shown seasonal variations depending on light exposure as have been reported in the literature.

Other limitations concern the instruments that were used for the ambulatory measurement of the light exposure. It should be noted that factors such as spatial sensitivity, spectral response, and absolute response influence the accuracy of the light measurements. Some authors have showed good correlations between instruments with sensors placed at the wrist and at the forehead [87].

However, contrary to this, Figueiro et al. [103] found large differences when measuring absolute levels of photopic illuminance with sensors positioned at the plane of the cornea and sensors placed at the wrist.

The instruments used in this study displayed some discrepancies between the photopic luminous efficiency function, V (*λ*), and the spectral response of the Actiwatch-L monitor and between a cosine distribution and the spatial distribution of the measuring instruments. Together with the risk of sleeve covering this may have resulted in an underestimation of the light exposure at the cornea during the winter when the angle between the sun and horizon are low, particularly at the high latitude where this study was carried out.

It should also be pointed out that the measurement in the present study reports on the lighting conditions to which this group of people were exposed. It is important to recognize that the light reaching the different photoreceptors on the retina also are influenced by factors such as pupil size and the ocular media [104, 105].

This study show the typical light exposure patterns during a normal work day for a group of subjects living in the south of Sweden. The light exposure patterns during weekends may look different as was shown by Hubalek et al. [88].

Results from different studies are not easily compared because of differences in sample, for example, age and occupation, in addition to seasonal differences in photoperiod and climate factors that have an influence on, for example, the time spent outdoors in daylight as well as the availability of daylight inside [86, 106, 107]. However, results from studies have shown that the daily duration of exposure to bright light (i.e., illuminance over 1000 lux) does not differ between different age groups within the range 18–57 years of age [78, 79].

From a practical point of view, the design of the study made it quite long and the participants were required to wear the portable instruments during 12 weeks across the year. An alternative design to investigate the seasonal variation in light exposure is choosing experiment weeks where the greatest differences between months occur, for example, December, February, Mars, April, July, August, September, and October. According to Young et al. [108], most onsets of SAD occur between the autumn equinox and mid-October. Fewer occasions may also allow longer measurement periods without being a too heavy load for the participants.

## Conclusions

A marked seasonal difference in daily exposure to light radiation was observed in this study. Concerning the circadian profiles of melatonin, the results showed a higher peak level during the dark winter period. On the other hand, no difference in the timing of the acrophase of salivary melatonin was observed. Furthermore, the subjects had higher concentration of melatonin at 07:00 during the winter in comparison to the spring, summer, and autumn. No seasonal variations in cortisol concentrations were observed.

During the last two decades, there have been great advances in the knowledge about light’s non-image forming effects on humans, showing that lighting conditions have a fundamental impact on health and psychological well-being in addition to supporting the visual needs of the users. Moreover, the increasing understanding of how various characteristics of the exposing light radiation affect the non-image forming effects of light offer improved possibilities for the development of appropriate lighting technology and designing lighting conditions in the built environment that supports the physiological and psychological needs of the users.

Most studies conducted have been carried out in the laboratory. However, concurrent with these advances, it is important to investigate the lighting conditions to which people are exposed to in their real working and living environments. There are relatively few field studies and only the most recent studies have investigated the spectral composition of the exposing light radiation. The complex interaction of different receptor systems, with different sensitivity functions, makes spectral composition an important characteristic to consider when investigating the non-image forming effects of light radiation [102, 109–113].

This study add to the results from other studies investigating light exposure in real working and living environments by reporting on diurnal and seasonal light exposure patterns for a group of Swedish office workers living at a high latitude of 56° N, a location at which no similar study have been carried out.
